# Simulation of transvascular transport of nanoparticles in tumor microenvironments for drug delivery applications

**DOI:** 10.1038/s41598-024-52292-0

**Published:** 2024-01-20

**Authors:** Fariha Shabbir, Amenah Abdul Mujeeb, Syed Faraz Jawed, Ali Haider Khan, Choudhary Sobhan Shakeel

**Affiliations:** 1 Department of Biomedical Engineering, Faculty of Engineering, Science, Technology and Management (ZUFESTM), Ziauddin University, Karachi, Pakistan; 2 Department of Biomedical Engineering, NED University of Engineering and Technology, Karachi, Pakistan

**Keywords:** Cancer, Computational biology and bioinformatics, Engineering, Nanoscience and technology

## Abstract

Nanomedicine is a promising approach for tumor therapy but penetration is challenged by complex tumor microenvironments. The purpose of this study is to design nanoparticles and analyze their transport in two abnormal microenvironments through a 2-D simulation. Employing a Computational Fluid Dynamics (CFD) approach, tumor vascular-interstitial models were initially simulated, and the impact of nanoparticles on the velocity profile and pressure gradient within the tumor microenvironment was observed. Through meticulous mesh analysis, it was determined that optimal outcomes were achieved using a quadrilateral meshing method for pancreatic tumor and a quad/tri meshing method for hepatic tumor. Results showed an increase in vessel diameter correlated with elevated blood flow velocity, reaching a maximum of 1.40 × 10^−3 m/s with an expanding cell gap. The simulation results for pressure distribution show that as vessel diameter increases, the velocity of nanoparticles in blood increases and decreases the pressure of blood. Intriguingly, distinct fluid flow patterns in pancreatic and hepatic tumors, emphasize how microenvironmental differences, specifically cell pore size, profoundly impact therapeutic agent transport, with implications for drug delivery strategies in cancer therapy. These simulation-based insights enable researchers to anticipate nanofluid behavior in realistic settings. Future work, incorporating immune cells, will enhance the understanding of nanoparticle efficiency in cancer therapy.

## Introduction

Angiogenesis has a non-remedial effect that leads to cancer. It is important to be inhibited before further proliferation of epithelial tissue cells which complicates a tumor’s environment. Angiogenesis is triggered by hypoxia^1^ and regulated with both activator and inhibitor molecules^2^. Unlike vasculogenesis, angiogenesis is strictly a pathological process leading to tumor formation and chronic inflammation^3^, while vasculogenesis occurs in embryonic development. Tumor development is dependent on an uninterrupted blood supply to satisfy nutritional demands. This facilitates vessel development from the prevailing microvasculature throughout tumor neovascularization. The process is managed by vascular endothelial growth factors (VEGF)^4^, which, broadly, are angiogenic factors^5^. The imbalance between anti-angiogenic and pro-angiogenic factors leads to initiates tumor angiogenesis^6^. An atypical angiogenesis method results in a microvascular system with a typical malformed vessel design and uneven flow patterns^7–9^. Hence, the development of a definite tumor microenvironment is achieved. It is the tumor microenvironment that influences the response of tumors to therapy or radiation treatment^10,11^. An aggressive response to medicine is exhibited by tumors due to a complex structured vasculature. Natural vascular epithelial tissue contains a hierarchical data structure. Arteries divide into arterioles, which later divide into thin-walled capillaries. Sleek muscle cells (SMCs) maintain vascular stability by wrapping around massive vessel epithelial tissue^12^. However, tumor epithelial tissue cells (TECs) are associated with uneven structures, ruffled edges, and long, weak protoplasm projections that stretch across the vessel lumen^13^.

Nanomedicine has become a well-liked possibility for tumor therapy^14,15^. Developments in nanomedicine are emerging, especially to utilize the Enhanced Permeability and Retention (EPR) factor during drug delivery^16,17^. The Enhanced Permeability and Retention (EPR) effect is the guiding principle of nanomedicine. It allows low relative molecular mass medication particles to concentrate in tissues and allows for vascular permeability in inflammation and growth channels. This influence has become the justification for the high quality of nanomedicine in cancer treatment. However, penetration in the abnormal microenvironment of a tumor for employment of nanoparticles as therapy is a major challenge. Nanoparticles have to be compelled to penetrate the tumor space via blood circulation, thereby being transported to various components of the tumor through this pathway. Then, nanoparticles are compelled to traverse the tumor walls of the vessel to pass through tissue and induce the treatment of the cell. Nanoparticle preparations and nanomedicine procedures have not yet achieved the required therapeutic result because the abnormal microenvironment of tumors has reduced the potency of nanoparticles in the tumor space. To address this challenge, it is imperative to comprehend the tumor tissue microenvironment through the application of computational fluid dynamics (CFD) modeling.

The abnormal setting of growth vessels and internal structure presents a serious hindrance to the transport of nanoparticles injected for cancer therapy. Other factors presented by the complex tumor microenvironment are heterogeneous blood flow, high interstitial fluid pressure, and dense extracellular matrix^18^. Additionally, the surface physicochemical properties of nanoparticles, such as the size and stiffness of nanoparticles, also affect their administration points and spatial distribution^19^. The passage of nanoparticles through vessel walls is influenced by two primary factors. The first factor pertains to the arrangement of the interstitial matrix in tumor vessels. The second factor involves the lack of a basement membrane in the irregular distribution of epithelium cells and within the blood vessels of tumors. This absence results in the development of highly permeable vessels within the interstitial matrix of the tumor.

Additionally, one of the most notable barriers that hinder the transport of nanoparticles in tumor tissues is the high extracellular pressure of the fluid. This can be caused by increased pressure of the fluid within the opening tumor’s matrix, which results in a low-pressure gradient^20^ between the walls of the vessels of tumors and the opening tumor’s matrix.

According to^21^, this nearly eliminates the pressure gradient. In an attempt to determine how interstitial fluid pressure (IFP) affects the transport of nanoparticles, Wu et al.^22^ coupled a continuous model growth of the tumor with a discrete angiogenesis model. This allowed them to study the hydrodynamics of the microenvironment and their effect on tumor growth. From the model, the simulation implies that increased interstitial fluid pressure in tumor tissues was caused by low lymphatic function and interstitial hydraulic conductivity. This also affected the nutrients and transvascular transport of anticancer drugs. A non-equilibrium thermodynamic model was proposed that supported mechanical and chemical principles. The simulation results showed that an increasing number of nanoparticles would decrease the permeation flux slowly.

Molecular dynamics (MD) simulations were employed to understand the structure and dynamics of PEGylated drug transporters, contributing to the in-silico design of highly efficient PEGylated drug delivery systems. Despite challenges, MD simulations prove valuable in determining optimal size, structure, and grafting density of PEG and carbon nanotubes^23^. Gao et al.^24^ offers a comprehensive review focusing on the theoretical simulation of active targeting of nanoparticles into tumor cells. Mathematical models consider microenvironmental factors and nanoparticle characteristics, discussing methods to overcome transport barriers for optimized treatment plans and improved therapeutic effects. Collectively, these studies advance the understanding of nanoparticle applications in tumor microenvironments, offering valuable insights for the design and optimization of targeted drug delivery systems.

This study presents tumor abnormal vascular-interstitial models that are rooted in the distinctive characteristics of tumor blood vessels. The model incorporates Computational Fluid Dynamics (CFD) simulations to investigate the transvascular transport of nanoparticles. In this study two specific tumor models were developed using ANSYS which represent pancreatic and hepatic microenvironments. Furthermore, nanoparticles were designed in this work and then CFD analysis techniques were applied to observe and record modifications in velocity and pressure gradients. The main objective of this study is to evaluate the effectiveness of nanoparticle transvascular transport within pancreatic and hepatocellular tumor microenvironments. This research endeavor delves into the intricate dynamics of nanoparticle transvascular transport within tumor abnormal vascular-interstitial models. Through the utilization of CFD simulations, the study achieves a realistic depiction of nanoparticle movement in blood vessels.

This study enhances the alignment of the simulation process with real-world scenarios, thereby elevating the accuracy of predictions. Notably, the study investigates the impact of influential factors like interstitial fluid pressure and blood flow velocity on nanoparticle transport. Additionally, it explores the nuanced effects of nanoparticle properties on transvascular transport, providing valuable insights for the design of nanoparticles optimized for drug delivery applications. This research can significantly contribute to a holistic understanding of nanoparticle behavior in tumor microenvironments, offering substantial guidance for the design of nanoparticles tailored to enhance therapeutic efficacy, particularly in the realm of cancer therapy.

## Method

Analysis of systematic literature review is employed in this study adheres to the PRISMA guidelines. An electronic search was conducted using the databases Science Direct, PubMed, and IEEE Xplore. “Nanoparticle”, “Tumor Microenvironment”, and “CFD” were the key terms used in the e-search. For scanning the databases with the provided keywords, the Boolean operators 'AND' and 'OR' were used. Only journal articles are included in the e-search since the year restriction was set between 2000 and 2023.

The initial electronic search technique yielded 692 research articles: 30 from Science Direct, 3 from PubMed, and 659 from IEEE Xplore. Only research publications published in journals were considered. There were 689 research articles left after the duplicates were removed; of these, 637 research articles were discarded following title and abstract reviews because they did not describe nanoparticle transport and tumor microenvironment and/or CFD technique. The remaining 52 publications were then subjected to in-depth evaluations, with each portion of each study paper extensively examined. Following the selection process, 16 pertinent research articles were included in this study. Table 1 presents these research publications, along with relevant information such as the primary author, year of publication, journal name, title, findings and limitations.

**Table 1:** Mesh quality report of all meshing methods for pancreatic tumors.

S. no	Inflation layer	Edge sizing (no. of divisions)	Minimum orthogonal quality	Maximum ortho skew	Maximum aspect ratio
Quadrilateral method					
1.	10	1	6.70978e−01	2.34456e−01	3.63928e + 00
2.	10	3	4.68226e−01	3.84909e−01	5.17224e + 00
3.	10	5	6.91148e−01	3.08852e−01	3.40166e + 00
4.	10	10	7.21825e−01	2.78175e−01	3.69574e + 00
5.	10	20	5.72693e−01	4.27307e−01	4.99995e + 00
6.	10	30	4.41131e−01	3.62436e−01	5.23601e + 00
7.	10	40	3.80658e−01	3.53791e−01	5.01286e + 00
8.	10	50	4.36652e−01	3.54350e−01	5.15501e + 00
9.	10	60	5.09027e−01	4.87246e−01	6.33131e + 00
10.	10	70	3.46548e−01	5.74376e−01	8.83890e + 00
11.	10	80	3.83203e−01	3.76913e−01	5.42193e + 00
12.	10	90	2.76609e−01	4.67791e−01	6.54355e + 00
13.	10	100	2.13825e−01	4.86299e−01	6.91370e + 00
Triangle method					
1.	10	1	5.69742e−01	2.50553e−01	4.22799e + 00
2.	10	3	5.79956e−01	2.37412e−01	4.10628e + 00
3.	10	5	5.36728e−01	2.35129e−01	4.17503e + 00
4.	10	10	4.88411e−01	3.40537e−01	5.00799e + 00
5.	10	20	4.86616e−01	3.11758e−01	4.75241e + 00
6.	10	30	4.64950e−01	3.36418e−01	4.96963e + 00
7.	10	40	4.56330e−01	3.34662e−01	4.97118e + 00
8.	10	50	4.36455e−01	3.39363e−01	5.03028e + 00
9.	10	60	3.42089e−01	3.50063e−01	5.29342e + 00
10.	10	70	3.81996e−01	3.72680e−01	5.35495e + 00
11.	10	80	3.58053e−01	3.42942e−01	5.17420e + 00
12.	10	90	3.93444e−01	3.52728e−01	5.15310e + 00
13.	10	100	3.67570e−01	3.88499e−01	5.52162e + 00
Multizone quad/tri method					
1.	10	1	2.14069e−01	7.27090e−01	1.42773e + 01
2.	10	3	5.44836e−01	3.52339e−01	5.22729e + 00
3.	10	5	6.16862e−01	2.53767e01	4.22557e + 00
4.	10	10	5.43791e−01	2.92747e−01	4.58842e + 00
5	10	20	5.23894e−01	3.82258e−01	5.57517e + 00
6.	10	30	3.14987e−01	6.06593e−01	9.61381e + 00
7.	10	40	3.55044e−01	5.60883e−01	8.48746e + 00
8.	10	50	2.54123e−01	6.78407e−01	1.19890e + 01
9.	10	60	1.97554e−01	7.47459e−01	1.54885e + 01
10.	10	70	1.41063e−01	8.18267e−01	2.17592e + 01
11.	10	80	1.06493e−01	8.62317e−01	2.88624e + 01
12.	10	90	1.69425e−01	7.82513e−01	1.80907e + 01
13.	10	100	1.42250e−01	8.16763e−01	2.15764e + 01

To collect relevant information from research publications for peer review, the following inclusion criteria were created. The review focused on studies examining nanoparticle transport, those employing the CFD technique for computational simulation, and investigations elucidating nanoparticle behavior within the tumor microenvironment. Additionally, eligibility criteria were constrained to original research articles written in English. This study excluded review papers, meta-analyses, systematic reviews, letters, thesis and dissertations, editorials, case reports, pre-prints or unpublished and cross-sectional studies. The results of systematic review which carried-out in this work are presented in the result section.

In this study, a two-dimensional tumor interstitial-vascular model was created based on the characteristics of the hepatic and pancreatic microenvironments, with the interstitial and vascular domains separated by a gap of cells. The simulation study for transvascular transport of nanoparticles in various tumor microenvironments were carried-out using the commercially available ANSYS 16.2 software package. Fluid flow modeling in Fluent was used to analyze the effects of velocity and pressure within the tumor microenvironment.

The multiple phases are mathematically handled as a continuum in the Euler-Euler technique. The idea of phasic volume fraction is developed because phase one volume cannot be filled by the volume of the other phase. The total of these volume fractions is 1. To produce a number of expressions with a similar structure for all phases, conservation equations are constructed for each phase. In the case of granular flows, these equations are solved by applying kinetic theory to provide constitutive relations. There are three distinct Euler-Euler multiphase models accessible in ANSYS fluid flow (Fluent): the model mixture, the volume of fluid (VOF) model, and the Eulerian model. The Eulerian multiphase model is used in this study. The Eulerian approach is the most complicated of the ANSYS Fluent multiphase models. For each phase, it solves a set of *n*th continuity and momentum equations. To establish coupling, the interphase exchange and pressure coefficients are employed. This coupling is treated differently depending on the kind of phases involved; non granular (fluid‒fluid) flows are handled differently than granular (fluid‒solid) flows. The characteristics of granular flows are derived from kinetic theory. The kind of mixture being represented also influences the momentum exchange equation between the phases. For modeling nanoparticles as a multiphase material, aluminum-solid as aluminum-oxide are assumed by setting the properties comparable to nanoparticles. Five boundary conditions are used in this model which are listed as: vascular-inlet, vascular-outlet, interstitial-inlet, interstitial-outlet, and wall. The phase-coupled simple solution approach is employed in the transport of nanoparticles and is based on an extension of the SIMPLE algorithm to multiphase flows. The Syamlal-O'Brien drag model is used for the fluid‒solid exchange coefficients; 200 iterations are assigned for the computation.

### Meshing

**Figure 1:**
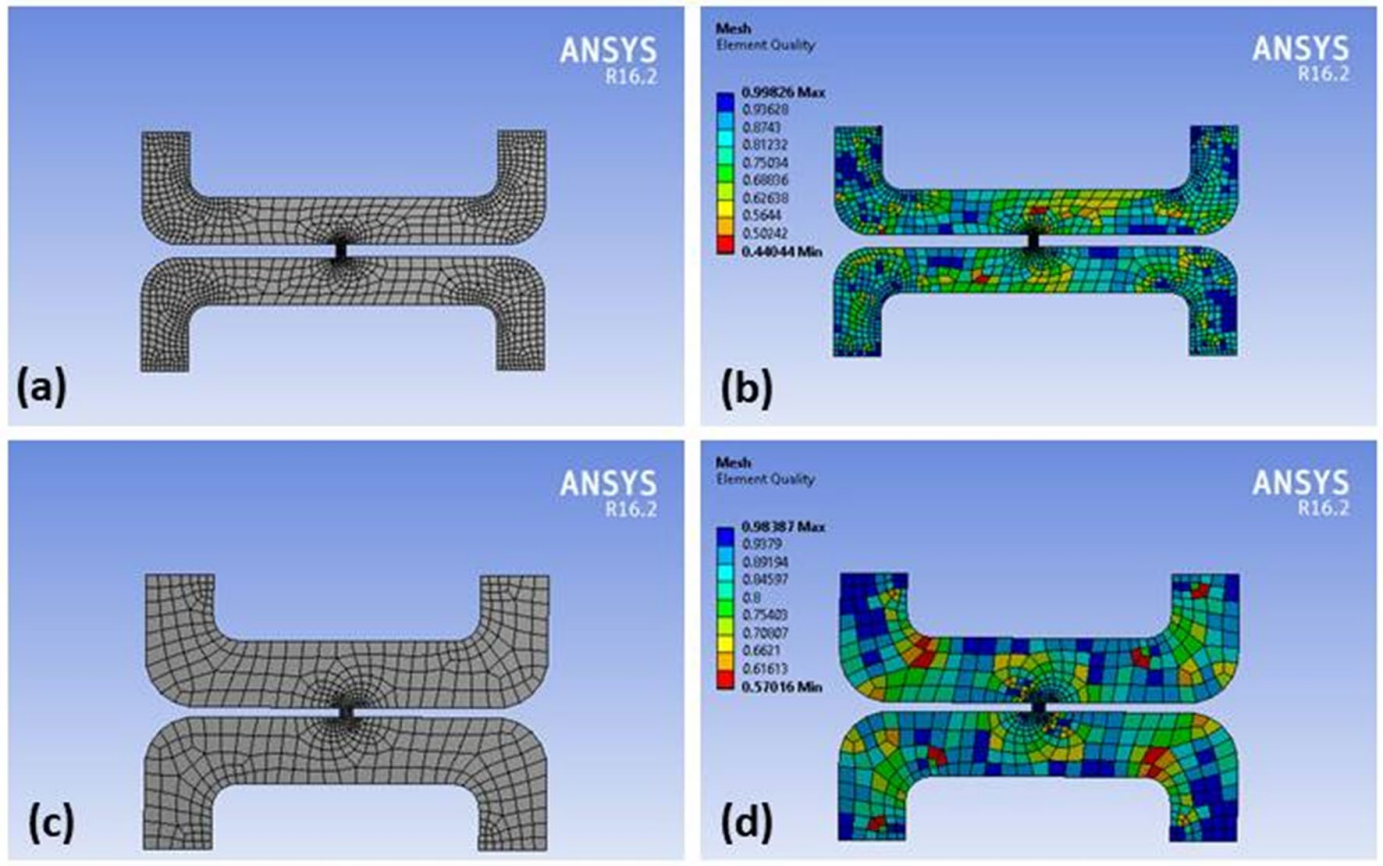
(**a**) Pancreatic Meshing Geometry (**b**) Pancreatic Mesh Element Quality (**c**) Hepatic Meshing Geometry (**d**) Hepatic Mesh Element Quality.

ANSYS Fluent package offers various meshing methods to create a high-quality mesh for a simulation. The structured meshing method generates a regular quadrilateral or hexahedral element grid that can capture complex geometries with fewer elements. The unstructured meshing method generates irregularly shaped triangular or tetrahedral elements that can capture complex geometries with more accuracy. The hybrid meshing method combines structured and unstructured meshing techniques to capture both advantages. It generates structured elements in the areas where the geometry is simple and unstructured elements in the areas where the geometry is complex. All three meshing methods are employed in this study to determine which method provides optimum results for the simulation study. The inflation layer is a boundary layer mesh that is created to capture the velocity gradient at the walls of the geometry. It is a thin layer of mesh that is created on top of the wall boundary and extends a short distance into the fluid domain. This layer is typically used for turbulent flow simulations, where the flow near the wall is strongly influenced by the viscosity of the fluid. The number of divisions or cells in a mesh is determined by the desired level of detail in the simulation. A higher number of cells typically results in a more accurate solution but can also increase the computational time. In order to ensure that the mesh size is appropriate for the simulation requirements, it is essential to control the total number of cells. ANSYS Fluent provides various tools to assess the quality of meshing as shown in Fig. 1. It can be noted from Fig. 1 that the mesh element quality of the pancreatic tumor and hepatic tumor geometries. Several crucial mesh quality indicators, including aspect ratio, skewness, and orthogonal quality, are employed to assess the overall quality of the mesh.

Tables 1 and 2 present the mesh quality report for the pancreatic and hepatic tumors, respectively. The orthogonal quality and orthogonal skew were assessed, with the former ranging from 0 to 1, where values closer to 0 correspond to low quality, and the latter ranging from 0 to 1, where values closer to 1 correspond to low quality. The pancreatic tumor's orthogonal quality was 7.21e−01, while that of the hepatic tumor was 8.53e−01. The orthogonal skew for the pancreatic and hepatic tumors was 2.78e−01 and 1.46e−01, respectively. The aspect ratio, which measures the mesh element's elongation, was 3.69e+00 and 2.68e+00 for the pancreatic and hepatic tumors, respectively. These values of metrics of quality of mesh are considered in this study.

**Table 2:** Mesh quality report of all meshing methods for hepatic tumors.

S. no	Inflation layer	Edge sizing (no. of divisions)	Minimum orthogonal quality	Maximum ortho skew	Maximum aspect ratio
Quadrilateral method					
1.	10	1	7.09983e−01	2.90017e−01	3.16512e + 00
2.	10	3	3.46695e−01	3.42890e−01	5.23329e + 00
3.	10	5	3.95717e−01	3.84271e−01	5.48057e + 00
4.	10	10	2.60411e−01	4.61865e−01	6.49894e + 00
5.	10	20	6.75170e−01	3.24830e−01	4.33212e + 00
6.	10	30	4.17821e−01	3.16603e−01	4.95500e + 00
7.	10	40	6.06465e−01	3.93535e−01	4.75598e + 00
8.	10	50	6.14901e−01	3.03246e−01	4.19209e + 00
9.	10	60	3.98599e−01	3.51848e−01	5.18258e + 00
10.	10	70	5.84080e−01	4.15920e−01	4.41844e + 00
11.	10	80	1.80842e−01	5.51221e−01	8.04498e + 00
12.	10	90	4.02358e−01	3.92881e−01	5.56609e + 00
13.	10	100	1.12827e−01	6.16848e−01	9.47902e + 00
Triangle method					
1.	10	1	5.96012e−01	2.28865e−01	4.07306e + 00
2.	10	3	4.86019e−01	2.87756e−01	4.57781e + 00
3.	10	5	5.98758e−01	2.54981e−01	4.23896e + 00
4.	10	10	4.22547e−01	3.65487e−01	5.27663e + 00
5	10	20	4.59066e−01	3.44322e−01	5.05481e + 00
6.	10	30	3.98670e−01	4.85326e−01	6.88245e + 00
7.	10	40	4.32809e−01	3.60680e−01	5.21533e + 00
8.	10	50	4.21860e−01	3.33373e−01	4.97646e + 00
9.	10	60	3.89261e−01	3.62228e−01	5.28316e + 00
10.	10	70	2.73254e−01	5.72784e−01	8.81790e + 00
11.	10	80	3.38461e−01	4.17780e−01	5.87842e + 00
12.	10	90	4.05995e−01	3.52010e−01	5.15735e + 00
13.	10	100	3.77564e−01	3.79752e−01	5.44021e + 00
Multizone quad/tri method					
1.	10	1	7.27081e−01	2.72919e−01	3.62542e + 00
2.	10	3	8.18073e−01	1.81927e−01	2.87604e + 00
3.	10	5	8.53479e−01	1.46521e−01	2.68959e + 00
4	10	10	8.17480e−01	1.82520e−01	2.38891e + 00
5.	10	20	7.81235e−01	2.18765e−01	3.10945e + 00
6.	10	30	8.14935e−01	1.85065e−01	2.50276e + 00
7.	10	40	7.37637e−01	2.62363e−01	3.04526e + 00
8.	10	50	8.36253e−01	1.63747e−01	2.75323e + 00
9.	10	60	7.43738e−01	2.56262e−01	3.12480e + 00
10.	10	70	7.58476e−01	2.41524e−01	2.92351e + 00
11.	10	80	7.93673e−01	2.06327e−01	3.11117e + 00
12.	10	90	7.44258e−01	2.55742e−01	3.38099e + 00
13.	10	100	7.84700e−01	2.15300e−01	2.90641e + 00

### Model of fluid flow

The flow of the fluid is considered steady and streamlined in this study, and because of that, we consider it to have the same density throughout its process^25^. Therefore, we related the two parameters of mass and momentum. For the flow in relation to mass and momentum, the equations are given below^41^:

1 $$\rho \nabla . \left(\overrightarrow{u }\right)=0$$

2 $$\rho \left(\overrightarrow{u }. \nabla \right)\overrightarrow{u }=\nabla .[-{\text{p}}\overrightarrow{I}\mu (\nabla \overrightarrow{u }\left(\nabla \overrightarrow{u }{)}^{T}\right]\overrightarrow{F},$$

where $\rho$ is density of fluid, $\overrightarrow{u}$ is velocity of fluid, ${\text{p}}$ is the pressure, $\mu$ is absolute viscosity, $\overrightarrow{I}$ is the unit tensor, and $\overrightarrow{F}$ is the external force.

### Transport model of nanoparticles

By calculating the expression for each particle, the model determines the path of particles floating in the fluid stream as they pass through the tumor aberrant interstitial vascular model. Newton's second law gives this equation as:

3 $$\frac{dy}{{dx}}\left( {m_{P} \vec{v}} \right) = \overrightarrow {{F_{t} }} ,$$

Where ${m}_{P}$ and $\overrightarrow{v}$ are related to the nanoparticle velocity and mass, respectively, and $\overrightarrow{{F}_{t}}$ represents the sum of the forces on that particle.

4 $$\overrightarrow{{F}_{t}}=\overrightarrow{{F}_{B}}+\overrightarrow{{F}_{D}}+\overrightarrow{{F}_{A}},$$

There are three components of the force acting on that particle that is suspended in fluid: where $\overrightarrow{{F}_{B}}$ is the Brownian force that is accountable for the haphazard movement of the suspended nanoparticle, $\overrightarrow{{F}_{D}}$ represents drag or opposing force, and $\overrightarrow{{F}_{A}}$ is the force acting in addition. Overall, research looks at the procedure of transvascular transport of nanoparticles at the mesoscopic position. As it is believed that nanoparticles remain unaffected by Brownian movement, the opposing force is the dominating force, which may be represented as:

5 $$\overrightarrow{{F}_{D}}=\frac{1}{{\tau }_{P}}{m}_{P}\left(\overrightarrow{u }-\overrightarrow{v}\right).$$

Here, ${\tau }_{P}$ represents the velocity time response of the nanoparticle, which is obtained as:

6 $${\tau }_{P}=\frac{{\rho }_{P}{d}_{P}^{2}}{18\mu },$$

where ${d}_{p}$ represents the diameter of the particle.

In addition, for tiny particles, it is preferred that a force named Saffman’s lift be considered an extra force $\overrightarrow{{F}_{A}}$, which can be represented as^41^:

7 $$\overrightarrow{{F}_{L}}=-18.2{r}_{P}^{2}\overrightarrow{{L}_{V}}\sqrt{\mu \rho \frac{\left|\overrightarrow{u}-\overrightarrow{v}\right|}{\left|\overrightarrow{{L}_{V}}\right|}},$$

8 $$\overrightarrow{{L}_{V}}=\left(\overrightarrow{u }-\overrightarrow{v}\right)\times \left[\nabla \times \left(\overrightarrow{u }-\overrightarrow{v}\right)\right],$$

${r}_{p}$ Implies the radius of the particle.

So,

9 $$\frac{d}{dt}\left({m}_{P}\overrightarrow{v}\right)=\overrightarrow{{F}_{D}}+\overrightarrow{{F}_{L}}.$$

In collision with the wall surface, the nanoparticles disperse slowly on the wall corresponding to the parameters, ensuring a shift in the direction of their motion. The dispersed rate of velocity is defined as follows:

10 $${v}_{t}=\left|\overrightarrow{{v}_{c}}\right|sin\theta$$

11 $${v}_{n}=\left|\overrightarrow{{v}_{c}}\right|cos\theta$$

where ${v}_{t}$ represents the velocity tangent to the particle, ${v}_{n}$ is the normal velocity, $\overrightarrow{{v}_{c}}$ is the velocity of the particle that collides with the wall’s surface, and $\theta$ is the angle between the surface normal and particle velocity of the wall.

### Special cases and parameters of the model

This study encompasses several crucial special cases to elucidate the intricacies of the tumor microenvironment and nanoparticle transvascular transport. A primary focus involves systematically varying the diameter of blood vessels, allowing for a detailed examination of its influence on the overall behavior of the tumor microenvironment, particularly on pressure and velocity gradients. Two distinct tumor models, representing pancreatic and hepatic tumors, are meticulously designed to facilitate a comparative analysis of microenvironments; the diameter of the blood vessel for the pancreatic tumor was set at 10 μm, and for the hepatic tumor was established at 20 μm. Additionally, the study delves into the nuanced impact of endothelial cell pore sizes, introducing variations (2 μm for pancreatic and 4 μm for hepatic tumors) that distinctly characterize the microenvironment of each tumor type. Notably, the study explores the influence of fluid dynamics by varying the initial velocities of the fluid inlets for the vascular and interstitial domains, with the former set at 1 mm/s and the latter at 0.1 mm/s^26^. The viscosity of blood is systematically controlled at 0.003 Pa s^27^, representing a specific and critical parameter affecting fluid behavior within the tumor microenvironment. Moreover, the study scrutinizes the transvascular transport potency of nanoparticles, the diameter of the nanoparticle was set at 0.2 μm^28^. The blood density was set at 1060 kg/m^3^^27^, as shown in Tables 3 and 4.

**Table 3:** Parameter values and ranges for pancreatic tumor.

Parameters of model	Value
Diameter of blood vessel	10 μm
Endothelial cell gap	2 μm
Blood density	1060 kg/m^3^
Interstitial fluid density	1000 kg/m^3^
Blood viscosity	0.003 Pa s
Interstitial fluid viscosity	0.0035 Pa s
Inlet fluid velocity of vascular domain	1 mm/s
Inlet fluid velocity of Interstitial domain	0.1 mm/s
Diameter of the nanoparticle	0.2 μm
Density of nanoparticle	800 kg/m^3^

**Table 4:** Parameter values and ranges for hepatic tumor.

Parameters of model	Value
Diameter of blood vessel	20 μm
Endothelial cell gap	4 μm
Blood density	1060 kg/m^3^
Interstitial fluid density	1000 kg/m^3^
Blood viscosity	0.003 Pa s
Interstitial fluid viscosity	0.0035 Pa s
Inlet fluid velocity of the vascular domain	1 mm/s
Inlet fluid velocity of Interstitial domain	0.1 mm/s
Diameter of the nanoparticle	0.2 μm
Density of nanoparticle	800 kg/m^3^

## Results

The results of systematic review are presented below in Table 5 which shows the state-of-the- art review and compare the various works that are carried-out in this scope.

**Table 5:** Comparative Analysis of Selected Studies for Literature Review.

S. no	Primary author	Year published	Title	Findings	Limitations
1.	Shamiul Sarkar Shishir^29^	2014	Blood flow dynamics in Cerebral Aneurysm—A CFD Simulation	The paper present findings related to the blood flow patterns, velocity distribution, pressure changes, or other relevant parameters within cerebral aneurysms. Results could help in identifying regions of high shear stress or turbulence that may be associated with an increased risk of aneurysm rupture	The paper discusses the limitations of the study, such as simplifications made in the computational model or assumptions taken. Improvement in model approach is suggested
2.	Mohammad Hassan Amiri^28^	2019	A 3-D numerical simulation of non-Newtonian blood flow through femoral artery bifurcation with a moderate arteriosclerosis: investigating Newtonian/non-Newtonian flow and its effects on elastic vessel walls	The study investigates various parameters, including velocity profiles, pressure distributions, and shear stress patterns within the femoral artery bifurcation. The effects of non-Newtonian blood behavior on these parameters, as well as its impact on the elastic properties of the vessel walls, are likely to be examined	Newtonian models for the blood flow do not lead to promising results at occluded areas and beyond them
3.	Yan Gao^30^	2020	Simulation study of the effects of interstitial fluid pressure and blood flow velocity on transvascular transport of nanoparticles in tumor microenvironment	The effect of blood flow rate on the transvascular transport of nanoparticles, but also clearly elaborates the mechanism and interaction of the effect of IFP and blood flow rate on the transvascular transport of nanoparticles	In the future, the true transvascular transport approach of nanoparticles can be explored, which lays an important foundation for the development of nanoparticles with higher transport efficiency
4.	Loke Kok Foong^31^	2020	Numerical simulation of blood flow inside an artery under applying constant heat flux using Newtonian and non-Newtonian approaches for biomedical engineering	Findings related to how heat is transferred within the artery under constant heat flux conditions. This could include temperature gradients, heat distribution patterns, and the overall thermal behavior of blood	The simulation likely involves simplifications and assumptions to make the study computationally feasible. These simplifications not fully capture the complexity of blood flow in real arteries
5.	Hamidreza Gharahi^32^	2016	Computational fluid dynamic simulation of human carotid artery bifurcation based on anatomy and volumetric blood flow rate measured with magnetic resonance imaging	Findings include information on shear stress distribution along the arterial walls and pressure gradients within the bifurcation. Understanding these factors is essential for assessing the risk of atherosclerosis and other cardiovascular diseases	Despite the patient-specific approach, certain simplifications and assumptions in the CFD model necessary for computational efficiency. These simplifications could impact the accuracy of the simulation
6.	Ahmed G. Rahma^33^	2022	Blood flow CFD simulation on a cerebral artery of a stroke patient	Findings indicate the relationship between shear stress patterns and the risk of stroke. Abnormal shear stress levels, particularly in specific regions, could be associated with increased vulnerability to stroke events	While the study focuses on blood flow patterns, stroke etiology is multifactorial, involving various risk factors. The CFD simulations may not capture all aspects contributing to stroke occurrence
7.	Davood Toghraie^34^	2020	Blood flow analysis inside different arteries using non-Newtonian Sisko model for application in biomedical engineering	The study is likely to reveal how the non-Newtonian Sisko model influences blood flow characteristics compared to traditional Newtonian models. Insights into shear-thinning behavior and other rheological aspects can inform the understanding of blood dynamics	While analyzing different arteries is valuable, the inherent variability in arterial characteristics across individuals may not be fully captured. The findings not be universally applicable to all arterial conditions
8.	Ryo Torii^35^	2001	Numerical simulation system for Blood flow in the cerebral artery using CT imaging data	Findings indicate how the specific geometry of the cerebral artery influences hemodynamics. Understanding these geometric influences is essential for assessing the risk of vascular diseases and optimizing treatment strategies. The study validates the accuracy of the numerical simulation results by comparing them against the original CT imaging data. This step ensures that the simulation model effectively represents the real-world conditions observed in the imaging data	The numerical simulation, while based on CT imaging data, still involve certain assumptions and simplifications for computational efficiency. The study might discuss the limitations of these assumptions and their potential impact on the results. The study acknowledges the limitation of not capturing temporal dynamics and fluctuations in blood flow
9.	Shu-Rong Yan^36^	2020	Analysis and management of laminar blood flow inside a cerebral blood vessel using a finite volume software program for biomedical engineering	The study likely provides insights into the laminar characteristics of blood flow, such as the formation of streamline patterns, velocity gradients, and the distribution of shear forces within cerebral vessels	The finite volume simulation likely involves certain assumptions and simplifications, which could affect the accuracy of the results
10	Loke Kok Foong^37^	2020	Numerical study for blood rheology inside an artery: The effects of stenosis and radius on the flow behavior	The study likely employs computational fluid dynamics (CFD) or similar numerical methods to simulate blood flow within an artery. This allows for a detailed analysis of fluid dynamics, considering the rheological properties of blood.it was found that possibility of turbulences and disease in blood stream in the stenosis artery with non-Newtonian blood is higher than that of Newtonian blood due to differences in viscous behavior and their reaction in exposure of applied shear stress via artery walls and stenosis	Sensitivity analyses be required to assess how variations in stenosis and radius parameters influence the numerical results. The study discusses the sensitivity of the model to different input parameters
11.	Shu-Rong Yan^38^	2020	Numerical investigation of non-Newtonian blood flow within an artery with cone shape of stenosis in various stenosis angles	It is reported that with increasing stenosis angles, blood flow temperature is decreased due to velocity enhancement.it is concluded that changing behavior of blood fluid from non-Newtonian to Newtonian behaviors can empower the capability of blood in thermal energy transfer inside human body vessels	Sensitivity analyses required to assess how variations in stenosis angles and other parameters influence the numerical results
12.	Liu Xingting^39^	2022	The thermal behavior of blood flow in the arteries with various radii and various stenosis angles using non-Newtonian Sisko model	It was found that artery radius and stenosis angles are effective in altering heat transfer rate so that increasing artery radius and decreasing stenosis angles of the artery causes the velocity of blood flow to be decreased	The findings may be specific to the chosen parameters and may not be universally applicable to all arterial conditions
13.	Muhammad Sabaruddin Ahmad Jamali^40^	2021	Effect of Different Types of Stenosis on Generalized Power Law Model of Blood Flow in a Bifurcated Artery	The study likely reveals insights into how different types of stenosis influence blood flow patterns within the bifurcated artery. Findings include variations in velocity profiles, shear stress distribution, and pressure gradients associated with different stenosis types	TYPE IV, stenosis located in the parent vessel, proximal and ostium of bifurcation. The type IV shows the most dangerous situation compare the others type so that in the future study that related to the stent need to carry out carefully
14.	Jaya Verma^41^	2023	Nanoparticle-mediated cancer cell therapy: basic science to clinical applications	Summarizes key findings related to the effectiveness of nanoparticle-mediated cancer cell therapy, both in preclinical studies and in clinical trials. This could include specific outcomes of clinical studies, improvements in treatment outcomes, or novel findings in the basic science realm	The review acknowledges potential limitations or obstacles in the translation of nanoparticle-mediated therapies, such as issues related to toxicity, scalability, or regulatory hurdles
15.	Pejman Shojaee^42^	2020	Effect of nanoparticle size, magnetic intensity, and tumor distance on the distribution of the magnetic nanoparticles in a heterogeneous tumor microenvironment	This study pioneers the exploration of capillaries' role in systemic magnetic nanoparticle (MNP) delivery, introducing a discrete model of idealized micro vessels	The study reveals limitations in systemic MNP delivery from tumor capillaries to tissue and underscores the impact of particle size on distribution
16.	Seyedali Seyedmirzaei Sarraf^43^	2021	Modeling and simulation of magnetic nanoparticles' trajectories through a tumorous and healthy microvasculature	This study employs computational 2D modeling and simulation in the COMSOL environment to analyze MNP trajectories. By considering differences between healthy and tumorous vascular networks, the study evaluates multiple geometry-close models under various magnetic field distributions. Capture efficiency, fallout coefficient, and MDT efficiency ratio are defined to assess the steering ability and potential side effects of magnetic drug targeting (MDT)	Assumptions regarding magnetic field distributions (MFD) and the selection of the optimal MFD based on particle tracing simulations introduce potential limitations in capturing the complexity of real-world magnetic environments

**Figure 2:**
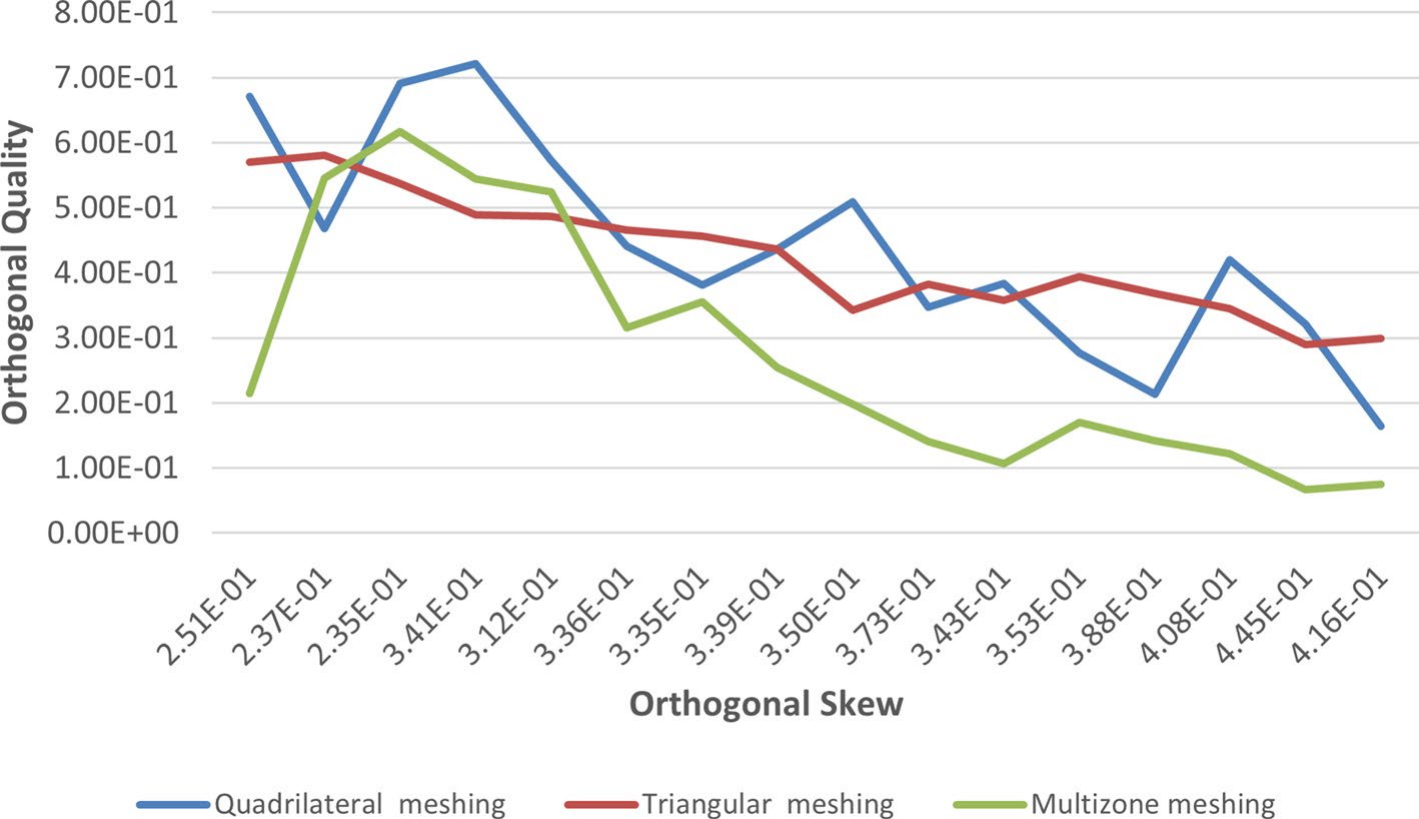
Meshing method analysis for pancreatic tumor.

**Figure 3:**
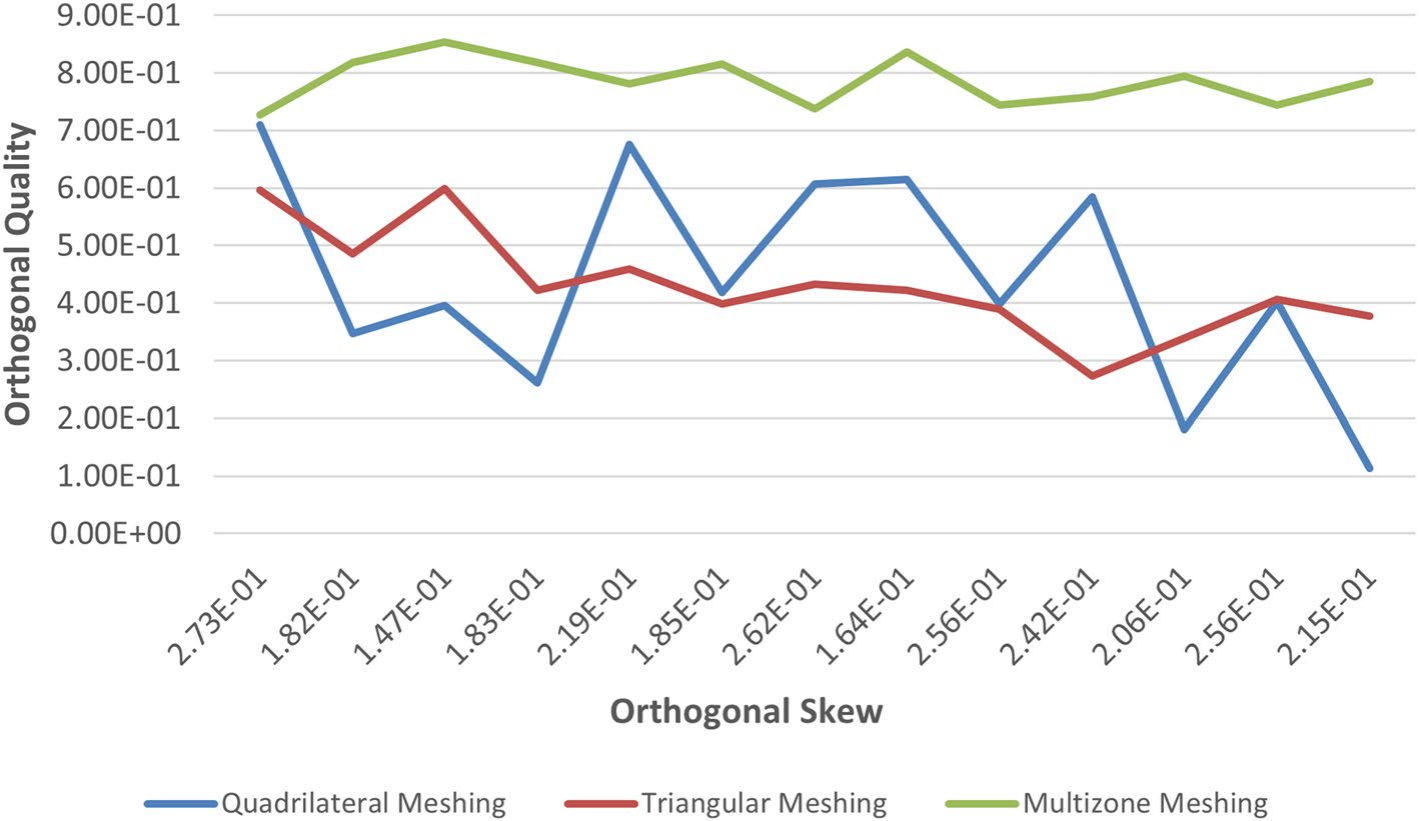
Meshing method analysis for hepatic tumor.

In this study, the quality of the mesh elements in the pancreatic and hepatic tumor microenvironments was assessed using several metrics, including element aspect ratio, skewness, and orthogonality. In Figs. 2 and 3, the analysis of the meshing method is presented. Please note that the computational strategy applied to understand the structural complexity of each tumor type. Specifically, Fig. 2 focuses on the meshing techniques employed for the pancreatic tumor, while Fig. 3 provides a detailed examination of the meshing process used for the hepatic tumor. The quadrilateral meshing method was used for the pancreatic tumor, while the multizone quad/tri meshing method was used for the hepatic tumor, as it provided optimal meshing quality.

**Figure 4:**
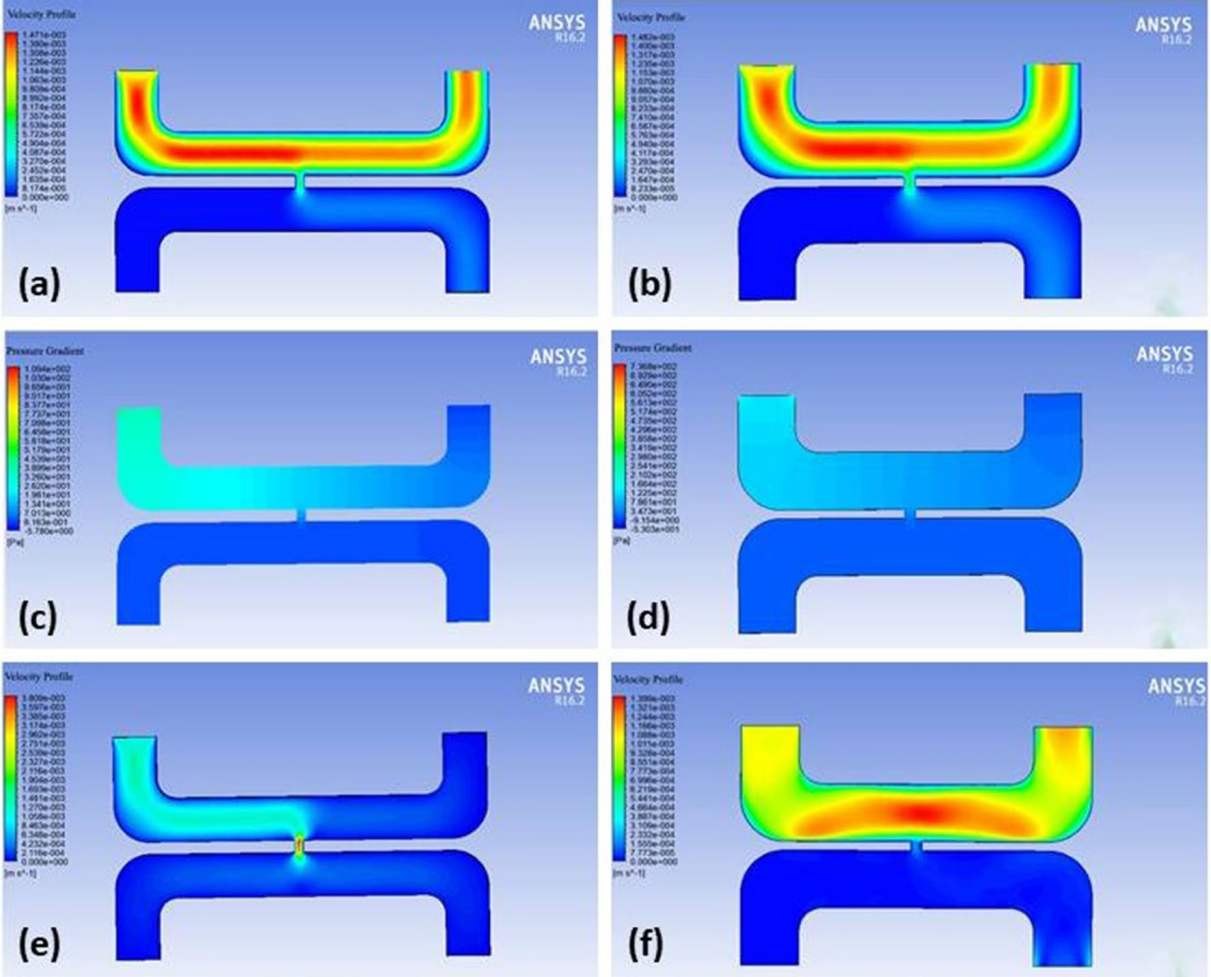
(**a**) Velocity profile of pancreatic tumor (**b**) Velocity profile of hepatic tumor (**c**) Pressure gradient of pancreatic tumor (**d**) Pressure gradient of hepatic tumor (**e**) Velocity profile of nanoparticle in pancreatic tumor (**f**) Velocity profile of nanoparticle in hepatic tumor.

The results of the simulation study are presented in Fig. 4, which displays the velocity distribution, pressure gradients, and nanoparticle velocity of nanoparticle transport efficiency. It can be noted from Fig. 4a,b that as the vessel diameter increased, the flow of nanoparticles in blood velocity also increased. This finding is evident in both the pancreatic tumor microenvironment and the hepatic tumor micro-environment. Notably, the velocity in the cell gap increased as the cell pore diameter increased, (Fig. 4a,b).

Simulation results for pressure distribution showed that as the vessel diameter increased from the pancreatic tumor vessel (Fig. 4c) to the hepatic vessel (Fig. 4d), the pressure decreased. This trend was also observed in the pressure within the cell pore. This can be further observed that as the endothelial cell gap increased from pancreatic Fig. 4c to hepatic Fig. 4d, the pressure in the cell pore also decreased. The simulation results for nanoparticle velocity in the pancreatic tumor showed that the velocity was maximum at the cell pore.

**Figure 5:**
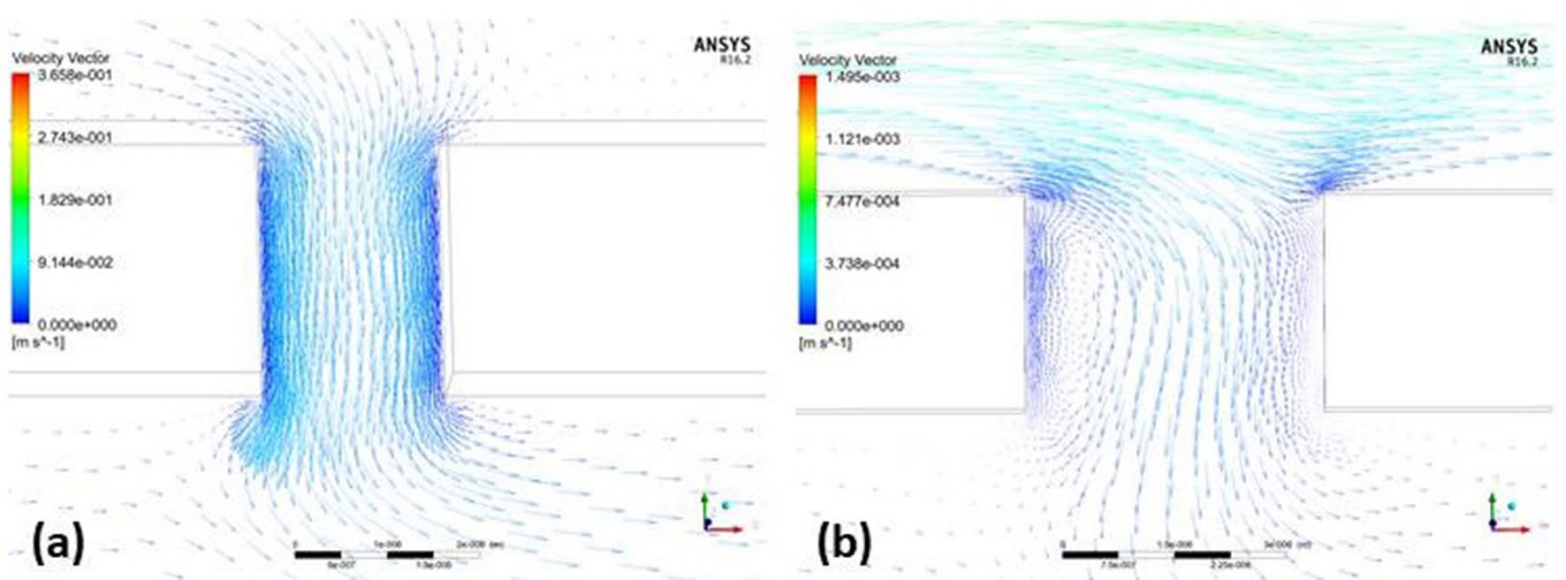
(**a**) Fluid velocity vector at the cell gap between vascular and interstitial domain in pancreatic tumor microenvironment (**b**) Fluid velocity vector at the cell gap between vascular and interstitial domain in hepatic tumor microenvironment.

Figure 5 presents distinctive fluid flow patterns in the tumor microenvironments studied. In the pancreatic tumor, characterized by a 2 μm cell pore between the vascular and interstitial domains, a majority of fluid velocity vectors successfully traverse the blood vessel and access the interstitial domain. The encounter with greater resistance results in shorter velocity vectors, and the smaller arrows illustrate a more confined fluid path due to tighter cell spaces (Fig. 5a). Conversely, the hepatic tumor, characterized by larger 4 μm cell pores, exhibits a smaller fraction of fluid velocity vectors reaching the interstitial. The majority of these vectors are transported towards the outlet of the blood vessel along with the blood flow. Longer velocity vectors in the hepatic tumor suggest less resistance, enabling fluid to travel greater distances within the tumor microenvironment, likely due to the larger cell pores facilitating a more streamlined flow (Fig. 5b). This observed variation underscores the profound impact of microenvironmental differences on fluid dynamics in tumors. Importantly, these findings emphasizing the pivotal role of unique tumor microenvironment characteristics, such as pore size, in influencing the efficiency of therapeutic agent transport and distribution across different tumor types.

**Figure 6:**
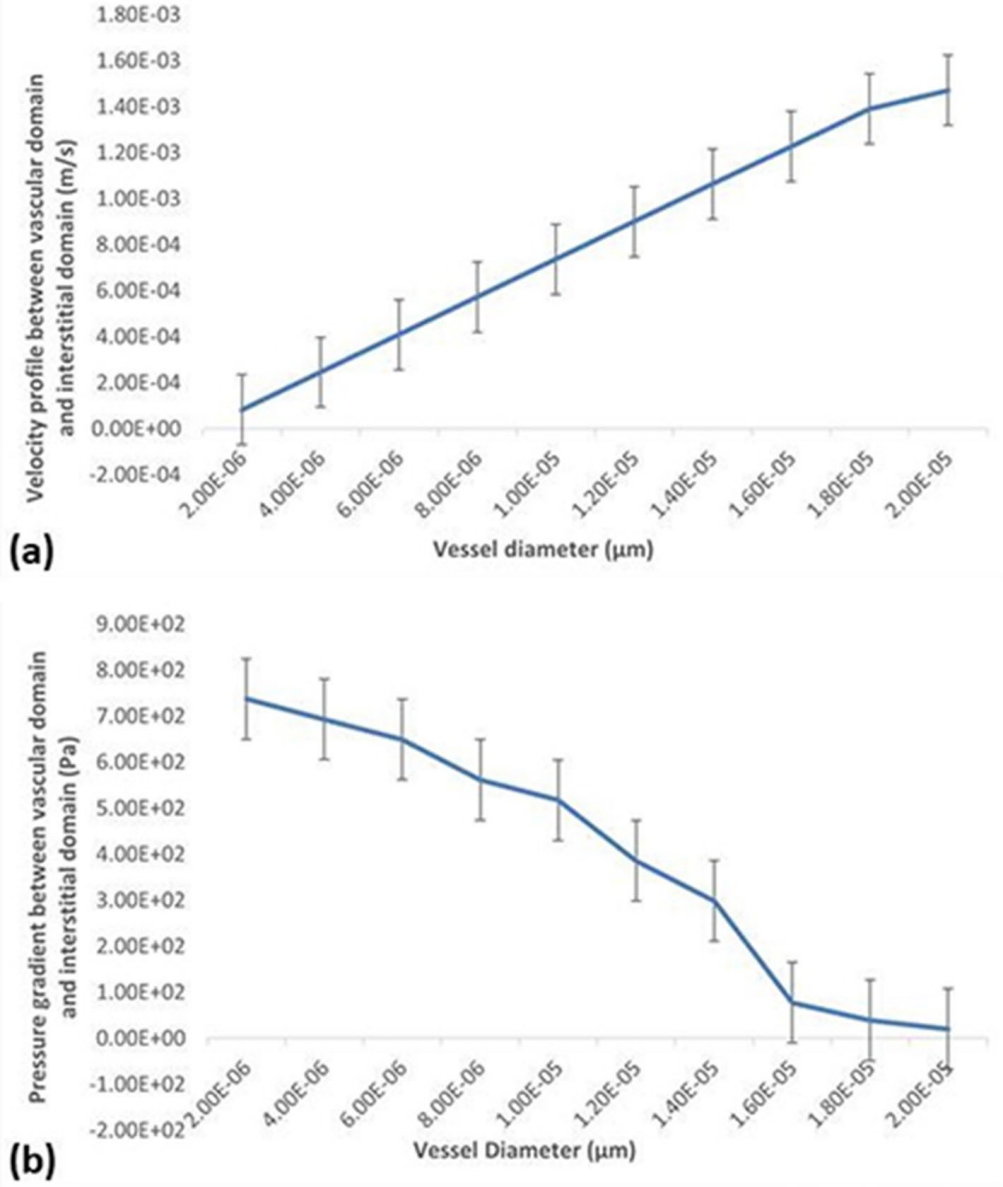
Trend graph of fluid (**a**) velocity profile and (**b**) pressure gradient at the blood vessel-interstitial tissue.

The analysis of the results presented in Fig. 6 revealed that as the blood vessel diameter and endothelial cell gap increased, the velocity of blood also increased (Fig. 6a), while the pressure of blood in vessels decreased (Fig. 6b). These findings suggest that as a benign tumor progresses to a malignant tumor, blood vessels may have a greater diameter than normal blood vessels, the findings are consistent with the conclusions drawn by Skinner et al.^44^.

These simulation results provide valuable insights into the mechanism of nanoparticle flow in blood for drug delivery, as well as for conducting nanoparticle-based drug targeting research. Specifically, this study sheds light on the influence of vasculature on the flow of nanoparticles in blood and may inform the design of more effective drug delivery strategies for the treatment of malignant tumors.

## Discussion

Microencapsulation and its derivatives, nanomedicines, are a popular area of research, particularly due to their nontoxic properties and timed-release mechanisms. Nanoparticle therapy is a more beneficial treatment for cancer than traditional therapies, which have long-term side effects. Nanoparticles, however, require more research for efficient drug delivery in the complex and abnormal microenvironment of tumors, where the flow of nanoparticles is seriously hindered by varying intra- and extracellular pressures as well as changing blood flow velocities.

The reported literature shows that the transvascular nanoparticle transport simulations were performed in tumor interstitial vascular models. The aim of this study was to observe the consequences of the tumor microenvironment and the rate of change in the flow of blood on transvascular nanoparticle transport. The second milestone of this study was to compare the effects of the tumor microenvironment and velocity of blood flow on the transport of nanoparticles within abnormal pancreatic and hepatic environments. The pancreatic and hepatic cellular environments were modeled for this purpose. The two tumor environments are differentiated by two parameters. These were vascular diameter and endothelial cell gap^10^. The tumor environments were varied, as hepatic carcinoma had twice the vascular diameter and endothelial cell gap as a pancreatic tumor. The blood density, initial velocity of the fluid inlet of the vascular domain and blood viscosity set in current model were according to the experimental work^30^. The quality of mesh elements is an essential consideration in simulations, as it can affect the accuracy and reliability of results. The meshing parameters used in this study were applicable for both tumors, indicating that the simulations were reliable and accurate.

It was discussed in^20^ that high extracellular fluid pressure will present difficulty for nanoparticle vascular transport, and is mostly caused by the high pressure of the interstitial matrix of the tumor. In this study, the intracellular pressure was focused, i.e., we changed the intracellular environment. By changing the vessel diameter and cell gap, the pressure gradient are indirectly altered and observed nanoparticle fluid flow in changing intracellular conditions.

According to the results of this study, velocity is at a maximum value at the center of the tumor vessel. Near the vessel boundary, the velocity is lower. Another notable result observed was that the pressure values are greatest at the vessel opening and at the cell gap. According to Figs. 4 and 6 of simulated models, it can also be deduced that the transport velocity of nanoparticles increased as the tumor vessel diameter increased. This, in particular, proved that as the vessel diameter increased, the pressure exerted by the nanoparticles in the blood decreased. As the abnormalities of a tumor microenvironment cause much complexity, it is difficult to accurately predict and define results. Modeling and simulating nanoparticle flow is challenging and still requires more improvement in methodology. In the future, this study will focus on designing nanoparticles with differing sizes, surface charges and morphology to determine the efficiency of nanoparticle transport through the tumor vasculature. Moreover, introducing immune cells in the models will aid in predicting nanoparticle accumulation in a tumor.

## Conclusion

In this study, the transvascular transport of nanoparticles was simulated in pancreatic and hepatic tumors, and the response of the abnormal tumor microenvironment to the process of this transport was analyzed. The enhanced permeability and retention (EPR) impact factor permits low relative molecular mass drug particles to accumulate in tissues and permits vascular permeability in inflammation sites and within tumor vessels. This impact has become the rationale for the quality of nanomedicine in tumor therapy. The diameter of the pancreatic blood vessel was set at 10 µm, and that of the hepatic blood vessel was set at 20 µm, while the flow of nanoparticles was simulated. With the CFD results, we determined that changing the blood vessel diameter affected the velocity and pressure of blood. The CFD results concluded that as the tumor blood vessel diameter increased, the flow of nanoparticles in blood velocity also increased, and the pressure gradient of blood decreased. In this study, the simulation model of the pancreatic tumor was a 10 μm diameter blood vessel, and the hepatic tumor was designed with a 20 μm diameter blood vessel. The results show that the velocity of nanoparticles in the blood vessels of hepatic tumors is higher than that in pancreatic tumors because the diameter of pancreatic tumor blood vessels is smaller. Therefore, the nanoparticles are delivered faster to hepatic tumors than to pancreatic tumors. However, when the benign tumor progresses toward a malignant tumor, its blood vessel diameter increases; therefore, the nanoparticle-based drug reaches faster in a malignant tumor than in a benign tumor. This study focuses on the delivery of nanoparticles in the abnormal tumor microenvironment and the targeting efficiency of nanoparticles.

## Future perspective

In future research, there is an opportunity to conduct more in-depth research into the authentic transvascular transport dynamics of nanoparticles. This exploration serves as a pivotal foundation for advancing the development of nanoparticles characterized by enhanced transport efficiency. The insights gained from a more nuanced understanding of the interplay between blood vessel parameters and nanoparticle kinetics, as demonstrated in this study, can contribute to the design and optimization of nanoparticles for improved targeted drug delivery in diverse tumor microenvironments.

## Data Availability

The dataset generated and analyzed during the current study are not publicly available but are available from the corresponding author on reasonable request.
